# The Texas Surgical Society

**Published:** 1915-04

**Authors:** F. E. Daniel


MRS. F. E. DANIEL.

### The Texas Surgical Society.

The first scientific session of the Texas Surgical Society, the new organization of Texas surgeons, will be held in San Antonio next October, the exact date not having been fixed as yet. But little publicity has been given to the fact of the organization of this society, which gives promise of becoming one of the strongest and ablest professional organizations in the State, the society preferring to go about its scientific studies without any blast of trumpets.

The president of the Texas Surgical Society is Dr. J. E. Thompson of Galveston; the first vice-president, Dr. J. H. Reuss of Cuero; the second vice-president, Dr. Frank Paschal of San Antonio; the secretary, Dr. B. W. Thorning of Houston; the treasurer, Dr. J. B. Smoot of Dallas, and the counsellors, Dr. John T. Moore of Houston and Dr. J. M. Doolittle of Dallas.

This list of officers in itself is sufficient to insure the high character of the society's membership and the purposes of the organization. The constitution and by-laws were adopted after careful and painstaking deliberation and with a view to enlisting as members only the ablest surgeons of the State, men of recognized merit and standing in the profession of surgery. The sole purpose of the society, aside from its social features, is the presentation and discussion of scientific papers on surgery and related subjects with a view to elevating the standards for those practicing surgery in the State and the general upbuilding of the profession.

The society admits to membership only those who have been engaged in surgical practice for five years or more with not less than 65 per cent of their practice surgical work. Members must be professionally and morally of good repute and standing, and unethical, unprofessional, or immoral conduct will subject them to dismissal. Among the practices that are condemned is "fee-splitting," which will not be tolerated. The society emphasizes its opposition to all questionable practices, and outlines its purposes and policies in such a way as to make it clear that it intends to so restrict its membership that it will at once become one of the most important professional organizations of the country. The high standing of its membership will be such that no one will be invited or will attempt to read a scientific paper before it without that paper having recognizable merit which can stand the test of discussion and criticism from the leading surgeons of Texas. To attempt to present a paper on a subject with which the writer is not thoroughly familiar would subject the author to ridicule if not contempt.

The membership will be not only restricted in character, but the number will be limited to 125, those constituting the membership at present being charter members. Surgeons desiring membership should make application, on blanks prepared especially for the purpose, to the secretary, Dr. B. W. Thorning, Houston, Texas, who will furnish blanks on request, and all applicants must have the endorsement of two members. All applications will be first considered by the board of counsellors, and if they receive favorable consideration will then be voted on by the society.

The TEXAS MEDICAL JOURNAL is glad to know of the formation of this society, believing that it will occupy a useful place in the profession and that it will have an immeasureable influence in advancing surgical practice.

